# Role of Butylphthalide in Immunity and Inflammation: Butylphthalide May Be a Potential Therapy for Anti-Inflammation and Immunoregulation

**DOI:** 10.1155/2022/7232457

**Published:** 2022-04-05

**Authors:** Yiliu Zhang, Yijun Ren, Xiqian Chen, Shuwen Deng, Wei Lu

**Affiliations:** Department of Neurology, The Second Xiangya Hospital of Central South University, Changsha, Hunan, China

## Abstract

Inflammation and immunity play an essential role in disease pathogenesis. 3-N-Butylphthalide (NBP), a group of compounds extracted from seeds of *Apium graveolens* (Chinese celery), has been demonstrated as an efficient and effective therapy for ischemic stroke. The amount of research on NBP protective effect is increasing at pace, such as microcircular reconstruction, alleviating inflammation, ameliorating brain edema and blood-brain barrier (BBB) damage, mitochondrial function protection, antiplatelet aggregation, antithrombosis, decreasing oxidative damage, and reducing neural cell apoptosis. There has been increasing research emphasizing the association between NBP and immunity and inflammation in the past few years. Hence, it is aimed at reviewing the related literature and summarizing the underlying anti-inflammatory and immunoregulatory function of NBP in various disorders.

## 1. Introduction

There is growing evidence that inflammation and immune response are critically involved in the initiation and severity of a series of other significant diseases. Neurological disorders, for example, acute ischemic stroke, activate inflammatory and immune cells within the central nervous systems (CNS) and induce the infiltration and accumulation of the inflammatory and immune cells from the peripheral system, which exacerbates the pathologies and worsens neurological prognosis [1]. Thus, the treatments with the ability to modulate inflammatory and immune responses can effectively prevent disease progression and minimize related disabilities.

3-N-Butylphthalide (NBP) is composed of optical isomers l-3-N-butylphthalide (l-NBP) isolated from seeds of *Apium graveolens* (Chinese celery), d-3-N-butylphthalide (d-NBP), and a synthesized compound, dl-3-N-butylphthalide (dl-NBP). NBP is oxidized by cytochrome P450 (P450) after oral administration. Moreover, hydroxylation of the n-butyl side chain and C-3 are involved in the primary metabolism process. Then, dl-NBP converts to four principal metabolites, including 10-keto-NBP (M2), 3-hydroxy-NBP (M3-1), 10-hydroxy-NBP (M3-2), and NBP-11-oic acid (M5-2), and finally is discharged mainly by the kidneys [2].

Furthermore, dl-NBP has been approved by the State Food and Drug Administration of China and introduced in the Chinese market as an anti-ischemic drug since 2002. Former clinical research and animal experiment concerning ischemic stroke have demonstrated that dl-NBP has multiple functions, including microcircular reconstruction [3], alleviating inflammation [4], ameliorating blood-brain barrier (BBB) damage [5], mitochondrial function protection [6, 7], antiplatelet aggregation, antithrombosis [8], decreasing oxidative damage [9], and reducing neural cell apoptosis [10]. Besides, NBP is an efficient and effective therapy for neurodegenerative diseases, brain edema, neural trauma, neurotoxicity, epilepsy, autoimmune diseases, and other nonneurologic conditions [11, 12].

## 2. The Potential Mechanism of NBP in Immune and Inflammatory Modulation

Some emerging experiments consider NBP as a novel agent for autoimmune disease treatment, such as idiopathic inflammatory myopathies (IIM) [13, 14] and multiple sclerosis (MS) [15]. Additionally, according to the current reports, a shift of macrophage/microglia polarization from proinflammatory M1 to anti-inflammatory M2 phenotype was observed in NBP-dependent ways [16, 17], indicating the possible underlying NBP's mechanism of inflammatory modulation and the potential of immune regulation. More explorations about the pathways via which NBP exerts a protective role are required. Therefore, based on existing evidence, we review and discuss the possible and potential NBP-mediated functions regarding regulating immunity and inflammation. The utility of NBP, targeting the signal molecules concerning the response to immune and inflammation, can be a promising therapeutic drug for inflammatory and immune-mediated diseases.

### 2.1. NBP and NF-*κ*B

Nuclear factor-kappa light chain enhancer of activated B cells (NF-*κ*B), as a family of evolutionarily conserved transcription factors, is well known in gene induction in a wide range of biological processes, including neurodegeneration, regulating cell growth and survival to immunity and inflammation [18–21]. Generally, the NF-*κ*B family of transcription factors consists of five members, p50, p52, p65 (RelA), c-Rel, and RelB. In resting cells, inhibitory protein I*κ*B*α* binds with NF-*κ*B, and the formation of complexes keeps NF-*κ*B as an inactive state in the cytoplasm. In canonical NF-*κ*B signaling pathways, phosphorylation, ubiquitination, and degradation of I*κ*B*α* in the proteasome then allow NF-*κ*B to translocate to the nucleus, bind to specific DNA binding sites, and initiate the transcription of target proinflammatory genes, upon stimulation such as lipopolysaccharide (LPS) [22, 23]. NF-*κ*B's specific binding regions have been identified in proinflammatory genes such as TNF-*α*, IL-1*β*, and IL-6. Accordingly, targeting the NF-*κ*B pathway is regarded as a therapeutic strategy against inflammatory disorders.

Accumulating research has shown that NBP modulates NF-*κ*B pathways. In current studies, NBP vastly reduced the NF-*κ*B protein level to alleviate inflammation in diverse animal models [24–27] and played a neuroprotective role in demyelination reduced by chronic cerebral hypoperfusion (CCH) [28] and ethidium bromide [29]. Numerous articles have demonstrated that the inactivation of the NF-*κ*B pathway is related to proinflammatory reactions in macrophages. Compared with the control group, LPS-stimulated RAW 264.7 macrophages have a higher protein concentration of NF-*κ*B-related proteins (p65 in the cell nucleus, phosphorylated-I*κ*B-*α*, phosphorylated-IKK-*α*/*β*) and lower cell cytosol protein concentration of p65, confirming that LPS induces the translocation of NF-*κ*B dimers from the cytosol to the nucleus to regulate macrophage/microglia properties. Conversely, pretreatment with AAL, a medicine extracted from Chinese medicinal plant, effectively inhibited this translocation and at the same time reduced production of TNF-*α* and IL-6 [30]. Similarly, previous studies revealed that administrations of NBP in rat models with cerebral ischemia reperfusion-induced brain injury [31] and with spinal cord injury [32] inhibited the expression of proinflammatory cytokines, including IL-6, IL-1*β*, and TNF-*α*, via reducing expression of TLR4 and NF-*κ*B (including p-NF-*κ*B, p-I*κ*B-*α*, and p-IKK-*α*). Furthermore, the polarization of macrophage/microglia is under control by NF-*κ*B. It has been reported that blocking NF-*κ*B on ovarian cancer cell conditioned media suppressed M1 macrophage-induced metastatic potential [33]. Another study showed that NBP remarkably suppressed the expression of nuclear p65 and reduced proinflammatory molecules in LPS-stimulated as well as MPPC-stimulated BV2 cells by Western blot analysis of nuclear and cytoplasmic fractions. Moreover, NBP have prevented the accumulation of nuclear p65 in response to LPS stimuli by immunofluorescence assay [34]. Based on the above results, we propose that NBP can regulate the polarization of macrophage/microglia via NF-*κ*B.

Besides the relationship with macrophage/microglia polarization, NF-*κ*B is associated with other immune cells. The functions of dendritic cells (DCs) depend on their maturation level. The maturation of DCs, in terms of upregulation of major histocompatibility complex and costimulatory molecules, is under control by activation of the NF-*κ*B pathway, especially the NF-*κ*B protein RelB [35, 36]. Inhibition of NF-*κ*B enables DC to induce Treg formation and Th2 polarization in vitro and in vivo [37, 38]. The indispensable roles of NF-*κ*B proteins in B cell development, maintenance, and function have been demonstrated [39]. Consequently, it has been presumed that NBP's function on innate or adaptive immunity cells is mediated through NF-*κ*B.

### 2.2. NBP and p38MAPK

The p38 mitogen-activated protein kinase (p38MAPK, termed here p38) is a vital signaling protein kinase that guides a signaling cascade to transmit extracellular signals to their intracellular targets. Abnormal activity and dysregulation of p38 have been shown to participate in the induction of pathologies such as inflammation [40], cancer [41], autoimmune diseases [42], Parkinson's disease [41], Alzheimer's disease [43], cardiac hypertrophy [44], and diabetes [45]. In many cases, p38 regulates inflammation and immunity, which contributes to the development of the diseases.

Some experimental results showed that NBP could regulate p38 expression. In the LPS-induced mouse model of Parkinson's disease (PD) [46] and rats of cerebral ischemia-reperfusion injury [5], phosphorylated-p38/p38 was significantly reduced following treatment with NBP. Contrary to the previous studies, NBP treatment promoted phosphorylated-p38/p38 in spinal cord injury (SCI) mice and BV2 cells [16]. Furthermore, there is a close association between macrophage/microglia polarization and p38 phosphorylation. For example, the p38 pathway is involved in microglia activation and positively affects microglia's proinflammatory secretory function in vivo and in vitro [46–49]. Another study showed that Gr-1(+) CD115(+) monocytes in tumor-bearing mice exhibited M2 characteristics. Conversely, LPS could transfer M2-type cells into M1 type through activating the P38 MAPK pathway, which, in turn, leads to the inhibition of the anti-inflammatory function of Gr-1(+) CD115(+) [50]. In BV2 cells and SCI mice, SB203580, a selective p38 pathway inhibitor, reversed the effect of NBP on inhibition of M1 marker expression and promotion of M2 marker expression [16], implying that NBP can enhance M2 polarization and inhibit M1 polarization in a p38-dependent way. Besides, p38 plays a significant role in macrophages to regulate the activity of transcription factors involved in inflammation response. Macrophages isolated from p38 *γ*/*δ* deficiency mice had lower reduced production of TNF-*α*, IL-1*β*, and IL-10, which demonstrated that p38 *γ/δ* are critical regulatory components of the innate immune response [51]. Environmental and cellular stresses stimulate p38 phosphorylation in macrophages [52], leading to the release of proinflammatory mediators, such as IL-1*β*, TNF-*α*, PGE2, and IL-12, as well as COX-2, IL-8, IL-6, IL-3, IL-2, and IL-1 from macrophages [53–56]. Inhibition of p38 by its specific inhibitor SB203580 significantly inhibited morphine-induced apoptosis and caspase-3 activation in BV2 cells [57]. Thus, it is reasonable to presume that NBP can regulate the p38 pathway somehow to influence macrophage/microglia activation, polarization, and subsequent expression of inflammatory mediators.

### 2.3. NBP and HIF-1*α*

Hypoxia-induced factor (HIF) is a transcription factor consisting of an alpha and beta subunit. There are three known alpha subunits (HIF-1*α*, HIF-2*α*, and HIF-3*α*) and three beta subunits (HIF-1*β*, HIF-2*β*, and HIF-3*β*, also known as ARNT1, ARNT2, and ARNT3). Except for the contributions to the cells' ability to adapt to changes in oxygen levels, angiogenesis, cell survival, invasion, and metastasis of the tumor, HIF is also related to the modulation of various immune cells, including macrophages, DCs, neutrophils, and T/B cells [58].

Researchers reported that dl-NBP treatment in a photochemical reaction-induced focal permanent middle cerebral artery occlusion (MCAO) model upregulated expressions of HIF-1*α* and VEGF [7]. The expression of HIF-1*α* was increased under chronic intermittent hypoxia hypercapnia (CIHH) exposure and was further expressed in rats with chronic NBP administration, which was consistent with the expression of Bcl-2/adenovirus E1B 19 kDa-interacting protein 3 (Bnip3), a known HIF-1*α* target protein [59]. HIF-1*α* expression in the nucleus was extraordinarily increased after rat brain microvessel endothelial cells (BMECs) were exposed to 2-hour oxygen-glucose deprivation (OGD) and 24-hour reperfusion and with NBP treatments [60, 61]. The above results suggest that NBP affects the expression of HIF-1*α* to a certain extent.

It is known that HIF-1*α* is involved in immune and inflammatory processes. In in vitro and in vivo inflammatory models, the deletion of HIF-1*α* dramatically decreased ATP levels in macrophages as well as reduced aggregation, motility, and macrophages' bacterial killing [62]. Overexpression of HIF-1*α* in macrophages promotes M1 polarization with a hyperinflammatory state [63–65], which is via upregulating expression of glycolysis and pentose phosphate pathway intermediates [64]. HIF-1*α* leads to T cells differentiating into Th17 through direct transcriptional activation of ROR*γ*t and subsequently p300 recruitment to the IL-17 promoter. Concurrently, HIF-1*α* reduces Treg development by binding with Foxp3 and targeting it for proteasomal degradation [66]. Similar proinflammatory characteristics for HIF-1*α* have been revealed in cell metabolism, differentiation, migration, and cell survival of DCs [67, 68], neutrophils [69, 70], and other immune cells [71]. It is reported that HIF-1*α*-dependent regulation of NF-*κ*B is directly involved in regulating neutrophil survival in hypoxia via a comparison between HIF-1*α* wild-type and gene knockdown murine neutrophils [69].

However, there are some controversies. NBP alleviates inflammation while it also inhibits HIF-1*α* with proinflammatory properties. On the one hand, the role of HIF-1*α* produced is different during various stages of the disease. Knockdown of HIF-1*α* during the early stage of Mycobacterium tuberculosis (MTB) infection resulted in a heightened disease state in these mice, while blocking HIF-1*α* during the late stage of MTB increased macrophage apoptosis and decreased bacillary loads [72]. The same results could be observed in sepsis [73]. Therefore, increasing expression of HIF-1*α* induced by NBP has a positive effect during the early stage of immune-mediated diseases. Accordingly, the data suggests that NBP may modulate immune cells (e.g., macrophage and neutrophil) and respond to inflammation through HIF-1*α*.

### 2.4. NBP and AMPK/SIRT1

AMP-activated protein kinase (AMPK), a serine/threonine kinase, is considered a metabolic sensor that maintains energy balance at the cellular and systemic levels [74]. Sirtuins (SIRT), as AMPK downstream molecules, belong to the class III histone deacetylase family and are classified into seven subtypes in mammals characterized by the same c.275-amino-acid core deacetylase domain and various N- and C-terminal domains [75]. As the most studied SIRT in mammals, SIRT1 is another nutrient sensor with widespread effects on metabolism and inflammation [76].

Many studies suggest that NBP affects SIRT1 expression. Min et al. confirmed that the neuroprotective effect of NBP under CIHH conditions might be caused by activating the SIRT1/PGC-1*α* signaling pathway [59]. At two weeks and four weeks after bilateral common carotid artery occlusion (2VO), NBP treatment suppressed inflammation, reduces demyelination, and promotes oligodendrocyte regeneration by reversing declining levels of AMPK/SIRT1 in CCH rats [28]. The expression of AMPK increased in the model of ischemic stroke after treatment with NBP [77].

Firstly, there is some evidence elucidating the role of AMPK in regulating immune cell metabolism and function. CD8+ T cells with deletion of AMPK*α*1 cannot revert to memory cells in metabolic dormancy [78]. Th cell development in response to infection requires AMPK*α*1 [79], which is in keeping with the experiments where the upregulation of AMPK increases the number of Treg cells for anti-inflammation [80]. Metabolically regulating immunity and inflammation of AMPK in natural killer (NK) cells was confirmed by the recent emerging report [81]. Pharmaceutical activation of AMPK, as a promising therapy, reduces the secretion of inflammatory markers (e.g., COX-2 and IL-1) [82, 83]. It is suggested that NBP can affect the differentiation of T cells and the functions of NK cells and finally regulate inflammation and immunity due to its similar effect on AMPK.

Secondly, inhibition of AMPK blocks autophagy via increasing mitochondrial reactive oxygen species (ROS) production, which is a way to reduce inflammation [84, 85]. Further, NBP pretreatment reduces the proinflammatory molecules and prevents oxidative damage by inhibiting the release of NO cells and the ROS production in BV-2 cells [34], in SH-SY5Y neuroblastoma cells [86] and in vitro animal models [5, 77, 87, 88], though increasing AMPK expression [77]. Thus, we speculate that NBP increases activation of AMPK and subsequently influences the expression of NO and ROS from immune cells to regulate inflammation.

Thirdly, the SIRT1-HIF1*α* axis guides the cytokines' production from the dendritic cell in the metabolically dependent ways, promotes the differentiation of CD4+ T cells, determines macrophage phenotype, and switches innate immune signals to adaptive immune responses [89, 90], which implies NBP can also produce the same immunomodulatory effect through the SIRT1-HIF1*α* axis.

Collectively, it indicates that NBP increases the expression of SIRT1 and AMPK to regulate immune and inflammation.

### 2.5. NBP and PI3K/Akt

Since the discovery of protein kinase B (PKB, also known as Akt) 25 years ago [91] and the identification of phosphatidylinositol 3-kinases (PI3K) as its upstream regulator [92], PI3K/Akt acts as a central node of many signaling pathways, such as immune modulation, tumor cell proliferation, and apoptosis.

Various experiments have shown that NBP affects the PI3K and Akt to play a protective role. P-Akt levels were decreased in the CCH 8-week group but activated in the NBP-treated group, coinciding with the results that NBP activated PI3K/Akt in oxygen-glucose deprivation/reperfusion (OGD/R) [93], the animal model of depression [94], MCAO rats [31], and bone marrow stem cells (BMSCs) [95]. Noticeably, expression of p-Akt instead of total Akt is dramatically increasing after NBP treatments. [96]. Similarly, in the study about OGD/R inducing cognitive impairment, there was no difference of total protein expressions of Akt and mTOR, Akt's downstream molecule, between any of the groups. In contrast, the vehicle group had a significantly higher p-Akt/t-Akt ratio and p-mTOR/t-mTOR ratio than the sham group [97], suggesting NBP mainly alters Akt phosphorylation.

It has been widely recognized that the PI3K/Akt pathway regulates and orchestrates the response to different metabolic and inflammatory signals in immune cells. Firstly, PI3K and Akt kinases' activation or overexpression suppresses macrophage activation [98]. Furthermore, activating the PI3K/Akt pathway is critically involved in restricting proinflammatory and promoting anti-inflammatory responses in TLR-stimulated macrophages [99]. The signals (such as TGF-*β* [100], IL-10 [101], and BMP-7 [102]) resulting in M2 macrophage polarization remarkably increase via PI3K/Akt. Suppression of Akt2 inhibits phagocytosis of opsonized beads from macrophages, demonstrating Akt activation appears essential for phagocytosis [103]. Akt and the downstream signaling pathway have been reported to produce the biological effects that enable neutrophils to better respond to viruses and microbial invasion [104]. As one of the well-known antiapoptotic molecules, Akt prolongs neutrophil survival time by directly controlling caspase-9 activity, phosphorylation of proapoptotic Bcl-2 family members (such as Bad) [105] and Ser184 phosphorylation of Bax [106]. It is accordant with the results that increasing p-Akt and decreasing expression of Bax are seen under NBP administration [14, 93].

Combined with the literature above, it is fair to assume that PI3K/Akt is another signaling pathway to exert NBP's effect on regulating macrophage activation, macrophage polarization, phagocytosis, and neutrophil longevity.

### 2.6. NBP and Nrf-2/HO-1

The nuclear factor erythroid 2-related factor 2 (Nrf2), a basic leucine zipper (bZip) transcription factor, protects a variety of tissues and cells from oxidative stress and inflammation through a variety of stage II detoxification and antioxidant enzymes (including NAD (P) H-H-1 (NQO1) and heme oxygenase-1 (HO-1)) mediated by the antioxidant response elements (ARE) [107, 108]. HO-1 is an ubiquitously expressed and essential cytoprotective enzyme that catalyzes the rate-limiting step in heme degradation, leading to equimolar quantities of carbon monoxide (CO), free iron, and biliverdin under pathologic conditions [109].

NBP is confirmed to have the effect of regulating Nrf2/HO-1. Zhao et al. found that PC12 neuronal cells exposed to OGD, as an in vitro model of ischemic stroke, significantly downregulated Nrf2 and HO-1, while NBP pretreatment significantly upregulated these genes [77]. The mRNA and protein expression of Nrf-2 and HO-1 in the NBP treatment group was dramatically increased at 24 hours after cerebral infarction compared with that in the control group [110], which was consistent with the effect of the NBP in a mouse model of amyotrophic lateral sclerosis [25]. Previous studies have shown that NBP increased Nrf2/HO-1 to inhibit atrial structural remodeling and finally prevent atrial fibrillation in heart failure rats [111].

Nrf2/HO-1 participates in regulating inflammatory immune cells through various mechanisms. As the primary anti-inflammatory and antioxidant enzymes are regulated by Nrf2 activation [112], HO-1 expression can affect the switch macrophages to M2 type in vitro [113, 114]. This role of HO-1 in regulating macrophage polarization has also been shown in experimental animal models of diabetes, Crohn's disease, hypertension, alcoholic liver disease, and bowel damage [114]. Moreover, pharmacologic induction of HO-1 inhibits human Th (T helper) and CD8+ cytotoxic T (TC) cell activation [115] and Treg cell function [116]. In keeping with these observations, biliverdin/bilirubin and CO (production of HO-1) inhibit Th cell activation [115, 117], induce apoptosis in Jurkat T cells [118], and suppress T cell-driven inflammatory pathologies, the rejection of transplanted organs [33], or autoimmune neuroinflammation [117]. Salutary effects of HO-1 are also exerted via sustaining tissue function and preventing endogenous proinflammatory ligands released from injured cells causing unfettered immune activation.

Accordingly, it is assumed that the protective effects of NBP are mediated essentially through immunoregulatory effects of Nrf-2/HO-1 exerted in cells of the innate or adaptive immune system.

### 2.7. NBP and Antioxidative Stress

Many immune-mediated inflammatory diseases are related to free radicals, which results in a high level of cellular oxidative stress and tissue injury. The death of cells driven by overreactive oxidative stress releases their intracellular components. These components act as proinflammatory and immunogenic agonists recognized by pattern recognition receptors (PRRs) expressed in immune cells, such as Mø and DCs [119]. In addition, oxidative-stress-dependent activation of transcription factors modulates the biosynthesis of antioxidant proteins and proinflammatory factors (such as NF-*κ*B and Nrf2 that could be regulated by NBP discussed above), and the activation is associated with inflammation and immune cells [120, 121]. Therefore, we presume the effect of NBP on modulating immune and inflammation might, at least in part, be exerted in such ways.

On the one hand, ROS is detrimental to cell structure by interacting with proteins and nucleic acids, especially lipids, resulting in the peroxidation of membrane phospholipids [122]. Severe metabolic disturbances and cell death happen as a consequence [123]. Superoxide dismutase (SOD) is a crucial antioxidative enzyme [124], while increased malondialdehyde (MDA) indicates oxidative damage of membranes, acting as an oxidative stress marker. NBP is shown to regulate the expression of these oxidative and antioxidant markers. The NBP treatment group has higher levels of both SOD and catalase (CAT), and lower levels of both MDA and proinflammatory cytokine (IL-1*β* and IL-6) were found in major depressive disorder (MDD) rats [26], diabetic rats [125], EAM guinea pigs [14], and BMSCs [95]. In cerebral ischemia-reperfusion injury rats, NBP reduced infarction area, cell apoptosis, blood-brain barrier destruction, and edema content through inhibition of ROS and MDA and via increasing activation of SOD [5]. Moreover, Nrf2 also plays an integral role in the antioxidative stress systems of cells. After being activated by oxidative stress, Nrf2 is transferred to the nucleus, and it binds to the ARE, finally elevating the expression of antioxidant genes and protecting cells from oxidative damage [126]. It has been shown that NBP could increase Nrf2 while reducing cellular oxidative stress. In the same study concerning MDD, researchers found the significantly upregulated level of Nrf2 in the nucleus, and the increasing trend of HO-1 and NQO-1, the Nrf2-downstream antioxidant genes, in NBP treatment [26], consistent with results from the experiment about OGD under NBP administration [77]. Thereby, these experiments suggest that NBP possesses the effect of antioxidation.

On the other hand, the average ROS/RNS, mainly produced by normal mitochondria, functions in healthy tissues to maintain normal physiological activities. Nevertheless, mitochondrial dysfunction may contribute to excessive and deregulated production of these molecules. Improper ROS, in turn, destroys mitochondrial inner membrane integrity, promotes mitochondrial depolarization, stems mitochondrial electron transfer chain, increases the opening of the mitochondrial permeability transition pore, and loses the intracellular calcium homeostasis [127–129]. It becomes a vicious circle and finally leads to intracellular oxidative stress and tissue damage. Thus, protecting mitochondria is another method to reduce oxidative stress and then meliorate inflammation caused by cell damage. NBP is proven to preserve normal cellular and mitochondrial function after OGD via stabling mitochondrial membrane potential (MMP), maintaining mitochondrial morphology, and boosting the activity of mitochondrial oxidative phosphorylation (OXPHOS) complexes (including complexes I-IV) and ATPase. NBP fixes the imbalance of protein that regulates mitochondrial fusion and division [77].

What is more, complex I, also named NADH-ubiquinone oxidoreductases, is linked with oxidative stress in mitochondria. Mutation leading to dysfunction of complex I has a positive effect on ROS production [130]. At the molecular level, NBP regulates the function of complex I to affect mitochondria and serve as an antioxidant agent mainly by competing for the sites (the 1,4-dihydronicotinamide adenine dinucleotide) [131]. (S)-ZJM-289, as a novel NO-releasing derivative of NBP, attenuated OGD/R-induced mitochondrial dysfunction with the noticeable restoration of mitochondrial complex I/IV activity. It also markedly decreased ATP level, ROS generation, and [Ca^2+^]_i_ accumulation in cortical neurons [132].

It has been reported that impaired mitochondrial biogenesis was alleviated by preserving mtDNA copy numbers [133]. TFAM has also been shown to maintain mtDNA and modulate the copy number [134]. NRF-1 is another crucial molecule in regulating energy supply and controlling mitochondrial biogenesis [135]. Tian et al. have found that NBP also significantly increased the contents of mitochondrial DNA (mtDNA) and mitochondrial biogenesis factors (NRF-1 and TFAM) after exposing cells to H_2_O_2_ [136], which further ascertains the function of NBP on promoting mitochondrial biogenesis. Therefore, NBP plays a role in stabilizing immunity and reducing inflammation by protecting the structure and function of mitochondria.

Given the above, besides the possible direct effects on immunity, NBP's roles include sustentation of tissue function and prevention of uncontrolled immune responses, which contribute to, to some extent, inflammation and immune modulation.

## 3. Therapeutic Potential of NBP in Inflammatory and Immune-Mediated Diseases

### 3.1. NBP in Idiopathic Inflammatory Myopathies

Idiopathic inflammatory myopathies (IIM) are a group of autoimmune diseases characterized by muscle injury and other organ systems' damage such as skin, lungs, and joints. Regardless of the different subtypes, the essential pathology of IIM is skeletal muscle infiltration by T cells, B cells, and macrophages [137]. Some emerging provided a basis for considering NBP as a novel agent for the IIM treatment. Compared with the control group, clinical manifestations and inflammatory cell infiltration of experimental autoimmune myositis (EAM, a common animal model mimicking IIM in humans) were dose-dependently ameliorated in guinea pigs treated with NBP, which is through improving the Ca2+-ATPase activity of the muscle's mitochondrial membrane and muscle's plasma membrane. Regarding the NBP effects on T cell-associated cytokines, NBP remarkably reduced the expression of IFN-*γ* mRNA in muscle tissues and significantly elevated Foxp3 and ROR*γ*t mRNA expression levels [13]. Additionally, NBP exerted a protective effect by improving the antioxidant enzyme activity, reducing oxidative damage, and decreasing the apoptotic muscle cells in an EAM model [14]. Therefore, more research is needed in the future to explore the therapeutic effect of NBP in IIM and its underlying mechanism.

### 3.2. NBP in Multiple Sclerosis

Multiple sclerosis (MS) is an autoimmune-mediated neurodegenerative disease characterized by inflammatory demyelination with axonal transection. Elevated expression of PGAM5 (the components of necroptosis complex [138]) and worse inflammation induced by experimental autoimmune encephalomyelitis (EAE, a common-used animal model of MS) were reversed by NBP administration. Moreover, reexpression of PGAM5 counteracted the protective effect of NBP on the pathogenesis of EAE, in accordance with the results seen in vitro. It is implicated that NBP suppresses microglial cell growth, necroptosis, and inflammatory factor release by regulating PGAM5 [15].

### 3.3. Future Perspectives

NF-*κ*B, as a critical role in the orchestration of the multifaceted inflammatory response, is active and exerts an effect in the production of inflammatory molecules in many inflammatory diseases, such as rheumatoid arthritis (RA), asthma, atherosclerosis, inflammatory bowel disease (IBD), or MS [22, 23]. It has been extensively studied that NF-*κ*B is a target in treating inflammatory diseases. For example, artemisinin and its derivatives inhibit NF-*κ*B by silencing these upstream pathways and/or directly binding to NF-*κ*B, which alleviates the severity of systemic lupus erythematosus (SLE), autoimmune encephalitis (AE), dermatitis, IBD, autoimmune hepatitis, and autoimmune thyroiditis [139]. NBP has been demonstrated to downregulate NF-*κ*B, implying NBP may be a potent and effective drug for the same autoimmune-mediated conditions via NF-*κ*B pathways.

P38 signal is a central hub in arthritis and inflammation of the liver, kidney, brain, and lung, and it acts as a critical player in inflammatory diseases mediated by immune cells such as macrophages [40, 42, 52]. Accumulating evidence under human clinical trials shows that p38 inhibitors are a promising therapeutic strategy to control inflammatory diseases, for example, RA and chronic obstructive pulmonary disease (COPD) [52]. Thus, NBP with the function of regulating p38 activity probably has the potential to treat RA, COPD, and other immune-mediated diseases.

Due to the significance of HIF-1*α*, AMPK/SIRT1, PI3K/Ak, and Nrf-2/HO-1 in the inflammatory response and immune cell responses, pharmacologically targeting these signal pathways has been considered a treatment of many different immune-mediated diseases, including sepsis, IBD, RA, cancer, and autoimmune encephalomyelitis [140–143]. Similarly, NBP might be an ideal approach for sepsis, IBD, RA, cancer, and autoimmune encephalomyelitis as its ability to target HIF-1*α*, AMPK/SIRT1, PI3K/Ak, and Nrf-2/HO-1.

## 4. Conclusion

**Figure 1:**
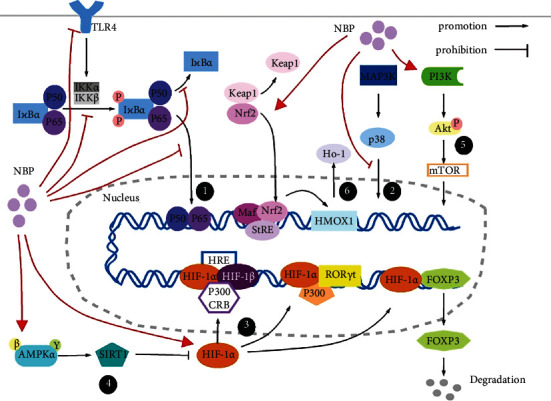
The potential mechanism concerning NBP's function on inflammation and immune.

Although NBP is considered a compound with proven efficacy in treating ischemic stroke and a growing body of research concerning NBP's effect on other diseases, there is a tremendous challenge of viable and effective transition from experimental to clinical practice. Besides, NBP's potential mechanisms on modulating immunity and inflammation for immune- and inflammatory-mediated disease remain unexplored. Based on these studies and data, we come to a novel perspective that NBP exerts anti-inflammation and immune regulation effects, at least partially, by modulating the signaling pathway discussed above (Figure 1) and alleviating oxidative stress. It potentially paves the way for a new strategy for immune-mediated diseases and inflammatory diseases to control immune responses. However, the dynamic changes of immune cells during the administration of NBP must be studied in much greater detail in the coming years. Thereby, further study will be needed to understand the precise molecular mechanisms of NBP concerning inflammation and immune response in the future.

The ways subsequently regulating immune cells are the following: ① upregulating proinflammatory factors (TNF-*α*, lL-1*β*, IL-6, etc.); promoting macrophages/microglia to express a proinflammatory phenotype (M1) and prohibiting the expression of the anti-inflammatory phenotype (M2); regulating DC development, survival, and cytokine production; modulating B lymphocyte survival during their differentiation and in their activation; prohibiting induction of Treg and Th2; ② increasing proinflammatory mediators (TNF-*α*, PGE2, IL-1*β*, IL-1, IL-2, IL-3, IL-6 IL-8, IL-12, COX-2, etc.); regulating macrophage/microglia polarization; increasing apoptosis of immune cells; ③ shifting macrophages/microglia toward M1 phenotype; modulating DC mature and immigration; regulating neutrophil extracellular trap formation and survival; differentiating and activating various T cells; reducing apoptosis of immune cells; ④ downregulating proinflammatory molecules (COX-2, IL-1, etc.); accommodating T cell differentiation towards the anti-inflammatory phenotype; determining macrophage/microglia polarization; decreasing the production of ROS/NO; reducing autophagy; ⑤ reducing apoptosis and prolonging immune cell survival time; promoting phagocytosis and macrophage/microglia polarization via inducing related regulators (TGF-*β*, IL-10, and BMP-7); ⑥ driving macrophage/microglia shift to M2 phenotype; prohibiting activation of Th and TC; regulating the function of Treg.

## Data Availability

No data were used to support this study.
